# Improving pain management in childhood acute otitis media in general practice: a cluster randomised controlled trial of a GP-targeted educational intervention

**DOI:** 10.3399/bjgp20X712589

**Published:** 2020-08-25

**Authors:** Rick T van Uum, Roderick P Venekamp, Nicolaas PA Zuithoff, Alies Sjoukes, Alma C van de Pol, Anne GM Schilder, Roger AMJ Damoiseaux

**Affiliations:** Julius Center for Health Sciences and Primary Care, University Medical Center Utrecht, Utrecht, the Netherlands.; Julius Center for Health Sciences and Primary Care, University Medical Center Utrecht, Utrecht, the Netherlands.; Julius Center for Health Sciences and Primary Care, University Medical Center Utrecht, Utrecht, the Netherlands.; Julius Center for Health Sciences and Primary Care, University Medical Center Utrecht, Utrecht, the Netherlands.; Julius Center for Health Sciences and Primary Care, University Medical Center Utrecht, Utrecht, the Netherlands.; University College London; National Institute for Health Research research professor evidENT, Ear Institute, University College London Hospitals Biomedical Research Centre, London, UK.; Julius Center for Health Sciences and Primary Care, University Medical Center Utrecht, Utrecht, the Netherlands.

**Keywords:** acute otitis media, child health, primary care, pain management, educational intervention

## Abstract

**Background:**

Pain management in acute otitis media (AOM) is often suboptimal, potentially leading to unnecessary discomfort, GP reconsultation, and antibiotic prescribing.

**Aim:**

To assess the effectiveness of a GP-targeted educational intervention to improve pain management in children with AOM.

**Design and setting:**

Pragmatic, cluster randomised controlled trial (RCT). GPs in 37 practices (intervention *n* = 19; control *n* = 18) across the Netherlands recruited 224 children with GP-confirmed AOM and ear pain (intervention *n* = 94; control *n* = 130) between February 2015 and May 2018.

**Method:**

GPs in practices allocated to the intervention group were trained (online and face-to-face) to discuss pain management with parents using an information leaflet, and prompted to prescribe weight-appropriate dosed paracetamol. Ibuprofen was additionally prescribed if pain control was still insufficient. GPs in the control group provided usual care.

**Results:**

Mean ear pain scores over the first 3 days were similar between groups (4.66 versus 4.36; adjusted mean difference = −0.05; 95% confidence intervals [CI] = −0.93 to 0.83), whereas analgesic use, in particular ibuprofen, was higher in the intervention group. The total number of antibiotic prescriptions during the 28-day follow-up was similar (mean rate 0.43 versus 0.47; adjusted rate ratio [aRR] 0.97; 95% CI = 0.68 to 1.38). Parents of children in the intervention group were more likely to reconsult for AOM-related complaints (mean rate 0.70 versus 0.41; aRR 1.73; 95% CI = 1.14 to 2.62).

**Conclusion:**

An intervention aimed at improving pain management for AOM increases analgesic use, particularly ibuprofen, but does not provide symptomatic benefit. GPs are advised to carefully weigh the potential benefits of ibuprofen against its possible harms.

## INTRODUCTION

Ear pain is the predominant symptom of childhood acute otitis media (AOM)^1^ and central to children’s and parents’ experience of the illness.^2^ Hence, symptom management with analgesics in adequate dosage (by body weight or age) in all children, and antibiotics in selected children, are the treatments of choice in most countries.^3^^–^^5^

In past decades, childhood AOM research has primarily focused on antibiotics, with only a few studies investigating the impact of symptomatic management with analgesics.^6^ Furthermore, current evidence suggests that in daily practice GPs very commonly give advice to use analgesics but fail to give parents explicit recommendations, particularly for children with AOM.^7^^,^^8^ As such, pain management may be suboptimal, and this may lead to unnecessary discomfort, doctor reconsultation, and antibiotic prescribing.

Conversely, there is some limited trial evidence of harm ensuing from the use of non-steroidal anti-inflammatory drugs (NSAIDs) — not just the well-known gastric and renal side effects, but direct harm on the progression of infections. The PIPS study^9^ found increased doctor reconsultations and complications, and the Internet Doctor trial found more prolonged illness, both as a result of NSAID prescribing.^10^ Recent trials in urinary tract infection (UTI)^11^^–^^13^ also suggest higher complication rates may result from treating UTI with NSAIDs, and there is mounting observational evidence that the use of NSAIDs for infections may result in harm.^14^^–^^17^ In children with AOM who experience insufficient pain relief with paracetamol alone (in weight-appropriate doses), the added benefit of ibuprofen remains unclear.

Therefore, a GP-targeted educational intervention was developed to improve pain management for children with AOM, and its effectiveness in reducing ear pain was trialled compared to usual care.

## METHOD

### Study setting, conduct, and participants

From February 2015 to May 2018, a pragmatic, cluster randomised controlled trial (RCT) was conducted in 37 GP practices (comprising a total of 81 GPs and 11 GP trainees) across the Netherlands: the Pain Intensity Monitoring in Paediatric Otitis Media (PIM-POM) study. The rationale and design of the study have been described extensively elsewhere.^18^

Children aged 6 months to 10 years with a GP-confirmed diagnosis of AOM (according to Dutch guidelines^4^) and ear pain were eligible, regardless of whether a child required immediate antibiotics or not. Children with grommets in place were excluded, as were children who had taken part in this study during a prior AOM episode and any siblings of those children. Also excluded were children with Down’s syndrome, craniofacial malformations, known immunodeficiency, liver failure, or renal insufficiency.

**Table:** How this fits in

Current guidelines emphasise the importance of symptomatic management with analgesics in children with acute otitis media (AOM). In daily practice, GPs frequently fail to give parents explicit recommendations. A tailored educational intervention, aiming to improve pain management, led to increased use of analgesics, particularly ibuprofen, but did not improve ear pain scores in children with AOM and was associated with an increase in GP reconsultations with subsequent antibiotic prescribing for AOM-related complaints. Considering this absence of a benefit to symptoms, GPs are advised to carefully weigh the benefits of using ibuprofen for this indication against its potential harms.

### Randomisation and blinding

The unit of randomisation was the GP practice; participating practices were randomly allocated to either the GP-targeted educational intervention or the usual care group, according to a computerised minimisation strategy^19^ with a random component of 30% designed by an independent statistician. Randomisation was stratified by GP practice size and age distribution of patients within each practice. Details on the randomisation procedure have been reported elsewhere.^18^

Due to the nature of the intervention, blinding was not possible. A cluster randomised design was adopted to avoid contamination between the groups. GPs and parents in the control group were not informed about the intervention, and were asked to participate in a study to monitor ear pain in children with AOM.

### Procedures

#### Intervention and comparator

GPs in practices allocated to the intervention group completed an online training module, met face-to-face with the study physician, used a parent information leaflet in their consultation, and were asked to prescribe (rather than recommend) analgesics.^18^ The intervention was developed according to guidance for complex interventions;^20^ as reported elsewhere.^18^

The online training module educated GPs about pain management in AOM through a combination of case-based learning, selfassessment with immediate feedback, reflection,^21^ and video demonstrations of effective communication techniques.^22^^,^^23^ GPs were trained to explicitly discuss pain management with parents using the parent information leaflet and prompted to prescribe weight-appropriate dosed paracetamol, and ibuprofen as add-on, if pain control via paracetamol appeared insufficient (see Box 1).^4^^,^^24^

**Box 1.** Recommended analgesic dosing

**Drug**	**Recommendation**	**Dosage**
Paracetamol	To be used in each patient	90 mg/kg/day, divided in three doses when given as suppositories, and for to six doses when given as syrup or tablets. This dosage is to be lowered to 60 mg/kg/day after 3 days
Ibuprofen	To be used additionally if pain control is insufficient despite optimally dosed paracetamol	20 mg/kg/day in three to four doses (syrup, tablets, or suppositories) for a maximum of 3 days

The parent information leaflet (see Supplementary Appendix S1) explained the importance of adequate pain management and included tables of weight appropriate dosing of paracetamol and ibuprofen. It also addressed common perceptions and misconceptions about the use of analgesics in children, and provided safety-netting advice.

When GPs had completed the online training module, a face-to-face meeting was scheduled with the study physician at the GP practice. In this session the main topics of the online training module were discussed, as well as potential barriers and facilitators to analgesic prescription to enhance adherence to the online module’s recommendations.

GPs in practices allocated to the control group did not receive this training and provided usual care. All participating GPs, regardless of trial group, completed an online otoscopy training module to standardise diagnosis. Taking a pragmatic approach, all other treatment decisions (for example, antibiotic prescribing) were left at the GP’s discretion.

#### Patient recruitment

GPs informed parents of potentially eligible children about the trial and obtained written informed consent. GPs completed non-recruitment logs, including reasons for non-recruitment of eligible patients.

#### Data collection

At inclusion, GPs recorded the child’s medical history and physical examination findings on a paper case report form (CRF). They provided parents with a paper study diary, in which parents reported daily symptoms and medication use (both over-the-counter [OTC] and prescription medication) for 14 days. Parents were asked to record their child’s ear pain intensity daily at the same time using the Wong-Baker FACES Pain Rating Scale (scores range from 0–10 with lower scores indicating less pain), an instrument proven reliable and valid in children aged ≥5 years,^25^^–^^28^ and applied in similar studies.^29^^–^^31^

At inclusion, parents reported whether the GP had advised analgesia in the same diary. At inclusion, and on day 14 and day 28 (end of follow-up), parents completed quality of life (QoL) questionnaires. Disease-specific QoL of the child was assessed with the Otitis Media-6 (OM-6), a 6-item questionnaire recording earrelated problems (scores range from 6–42 with lower scores indicating better QoL).^32^ Generic QoL of the parents was assessed with the EuroQol EQ-5D Visual Analogue Scale (EQ-5D VAS), scores range from 0–100 with higher score indicating better QoL.^33^^,^^34^ On day 28, parents completed a productivity loss questionnaire using an adapted version of the iMTA Productivity Cost Questionnaire.^35^

The study physician contacted parents by phone on day 3 to optimise compliance and ask parents about the ear pain scores and analgesic use over the first 3 days. After 28 days, the same study physician contacted parents by phone or email with a reminder to return the completed diary by mail. For participants from whom no diary was received, the primary outcome data captured by phone on day 3 was used. During the 28-day follow-up period, the study physician visited the GP practices on a regular basis to collect data on antibiotic prescribing, reconsultations, hospital admissions, and specialist referrals from the children’s electronic medical records in a paper CRF.

All available data were merged into an electronic database by an independent data manager. Two researchers performed source data verification: one checked 100%, and the other independently checked a random sample of 10% for accuracy.

#### Outcomes

The primary outcome of interest was the parent-reported mean ear pain score over the first 3 days. Secondary outcomes were:

- the total numbers of days with ear pain and fever;
- proportion of children with ear pain at 24 hours, 72 hours, and 7 days;
- number of reconsultations (practice visits and telephone consultations) and antibiotic prescriptions because of AOM;
- (serious) adverse events of analgesics;
- complications of AOM (for example, meningitis or mastoiditis);
- days lost from work for parents;
- days lost from day-care or school for children during 28 days of follow-up; and
- QoL scores of the child (OM-6 at day 28) and the parents (EQ-5D VAS at day 14).

#### Sample size calculation

In a previous childhood AOM trial, the mean ear pain score on days 1–3 was 3.7 (standard deviation = 2.6).^36^ To detect a clinically relevant 25% reduction with 80% power at a 5% significance level, a minimum of 66 children per group was needed. With an inflation factor of 1.7 for the cluster design, assuming a cluster size of 15 children and an intra-class correlation coefficient of 0.05 (for GP practice level),^37^^,^^38^ 115 children per group were needed. To allow for 10% attrition,^39^^,^^40^ the aim was to include 125 children in each group.

#### Statistical analysis

A statistical analysis plan was predefined and published prior to analysis.^18^ Data analysts were not blinded to treatment allocation. All analyses were performed according to the intention-to-treat analysis principle. For descriptive purposes, mean parent-reported ear pain scores (observed values) for each treatment arm were plotted over time using bar charts. The primary outcome was analysed with a linear mixed model with a residual covariance (that is, generalised estimating equation type) matrix for repeated measurements. In an initial model, a random intercept for GP practice was included to account for cluster randomisation. The intercept was close to zero, and therefore excluded from subsequent analyses. In crude analysis, treatment group and time were included in the model. In adjusted analysis, pre-specified confounders and prognostic baseline variables were added to the crude model.^18^ The validity of the model (that is, normality and homoscedasticity) was evaluated by assessing residuals. The comparison between treatment groups was reported as differences in means with 95% confidence intervals (CIs).

In sensitivity analyses, missing baseline (*n* = 10–105, depending on variable) and outcome data (*n* = 14) were imputed for using the IBM SPSS Statistics (version 25.0) multiple imputation function.^41^ The data were imputed 10 times and the analyses were performed in each imputed dataset. The results were subsequently pooled using Rubin’s rules.^42^

For the secondary outcomes, Poisson regression analyses were used for count variables, mixed logistic regression analyses used for dichotomous variables, and multiple linear regression analyses used for continuous variables. For these analyses, the comparison between treatment groups were reported as rate ratios, odds ratios, and mean differences (MDs) respectively; all with 95% CIs. As per analysis for the primary outcome, a random intercept for GP practice was included in each secondary outcome model primarily to account for clustering. As only minimal clustering effects were observed for some of the secondary outcomes, which affected neither estimates nor precision, the random intercept was excluded from the final analyses of all secondary outcomes. Furthermore, since complications of AOM and (serious) adverse events of analgesics were very rare, regression analyses were not performed for these outcomes. All statistical analyses were performed with IBM SPSS Statistics (version 25.0), and residual analyses for linear mixed models were performed with SAS (version 9.4).

## RESULTS

### Participants

**Figure 1.**
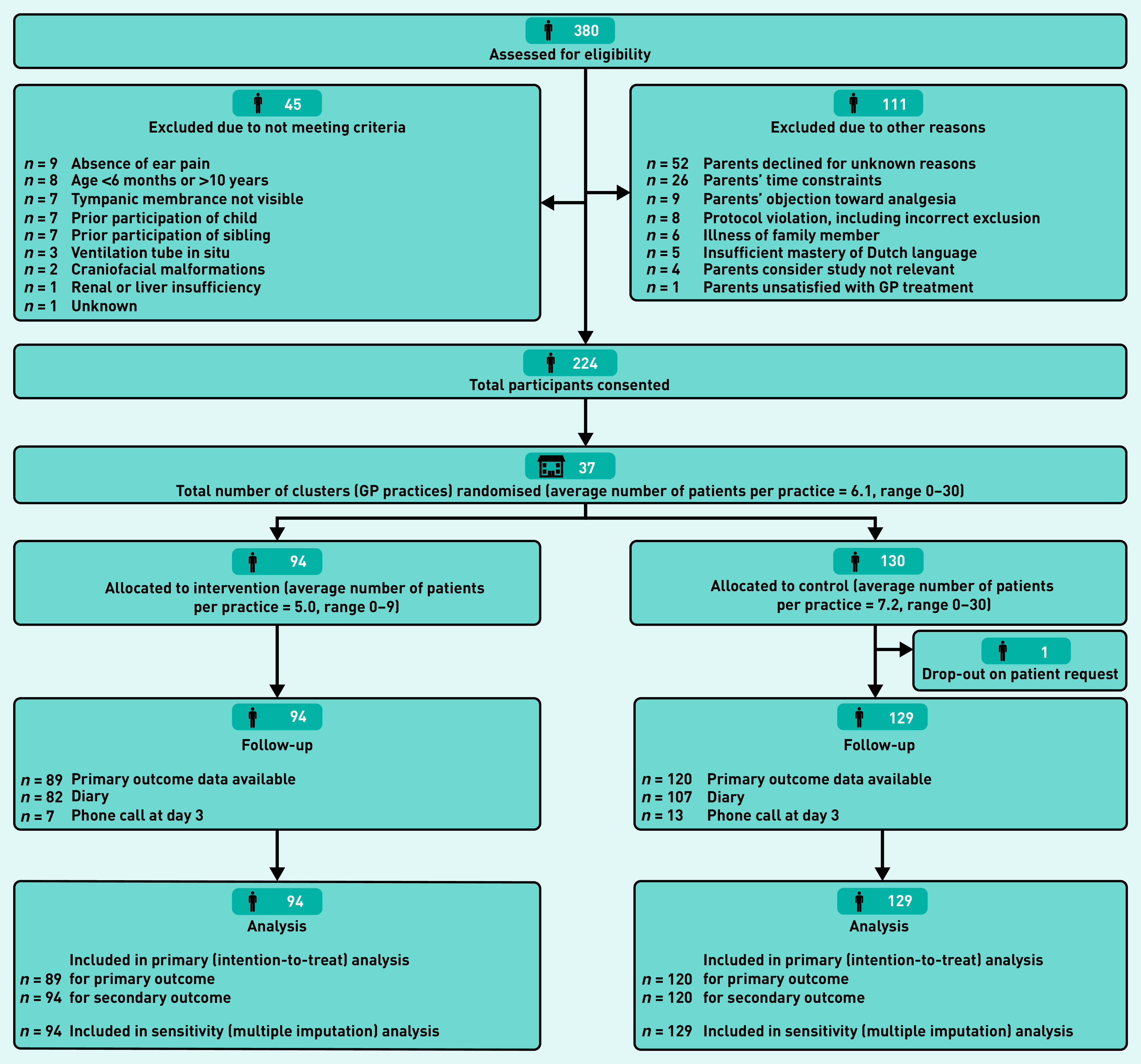
***Consort diagram.***

Figure 1 illustrates the flow of children through the trial. Of the 380 children assessed for eligibility, 224 participated in the trial (intervention *n* = 94; control *n* = 130). One child in the control group dropped out due to illness of the parent. Diary data were missing for 20 (intervention *n* = 7, control *n* = 13) of the remaining 223 children. For six of these children primary outcome data were obtained during the phone call on day 3; therefore, the data were available for 209 participants (93.3%) in total.

Table 1 summarises the baseline characteristics of participating GP practices, GPs, and included children. The baseline characteristics of GP practices, individual GPs, as well as children were generally well-balanced. However, children in the intervention group had a slightly shorter duration of ear pain prior to the index consultation, less bulging of the tympanic membrane, and fewer bilateral AOM, but more often a history of recurrent AOM, previous ear, nose, and throat (ENT) surgery, and atopic constitution.

**Table 1.** Baseline characteristics

**Characteristics**	**Intervention (*N* = 48; 19 practices)**	**Usual care (*N*= 44; 18 practices)**
**GP practices**		
Geographical location, *n* (%)		
Urban	10 (52.6)	8 (44.4)
Semi-rural	4 (21.1)	6 (33.3)
Rural	5 (26.3)	4 (22.2)
Practice population, mean ± SD	4068 ± 2275	4402 ± 2850
Percentage young children, mean ± SD	10.9 ± 2.4	11.5 ± 3.1

**GPs**		
Sex, male, *n* (%)	27 (56.3)	22 (50.0)
Age, years, mean ± SD	50 ± 11	47 ± 11
Experience, years, mean ± SD	19 ± 11	17 ± 11


	**Intervention**	**Usual care**
	**(*N* = 94; 19 practices)**	**(*N*= 129; 18 practices)**

**Children**		
Age, months, median (IQR)	38 (13–62)	43 (20–66)
Sex, male, *n* (%)	54 (57.4)	66 (51.2)
Weight, kg, median (IQR)^a^	15.0 (10.0–20.0)	14.4 (9.8–18.9)
Daycare or school attendance, *n* (%)^a^	38 (46.9)	48 (37.2)

**Parental educational level (higher), *n* (%)^a^**		
Mother	44 (55.7)	55 (53.4)
Father	33 (48.5)	52 (54.7)

**Medical history, *n* (%)**		
Recurrent AOM	14 (14.9)	12 (9.3)
Recurrent URTI	13 (13.8)	11 (8.5)
Previous ENT surgery	15 (16.0)	8 (6.2)
Atopic constitution	16 (17.0)	8 (6.2)

**Risk factors, *n* (%)^a^**		
Pneumococcal vaccination	79 (97.5)	106 (97.2)
Household smoking	4 (4.9)	4 (3.7)

**Symptoms prior to consultation (parent-reported)**		
Ear pain, *n* (%)^a^	75 (90.4)	91 (84.3)
Days, *n*, median (IQR)	2 (0.5–3.5)	3 (1–5)
Otorrhea, *n* (%)^a^	12 (15.4)	16 (16.0)
Days, *n*, median (IQR)	2 (0–5)	2 (0–4.5)
Fever, *n* (%)^a^	50 (60.2)	76 (67.3)
Days, *n*, median (IQR)	2 (1–3)	3 (2–4)

**Physical examination, *n* (%)**		
Temperature, °C, mean ± SD^a^	37.6 ± 0.9	37.9 ± 1.1
Ill appearance	18 (19.6)	25 (20.3)
Unilateral AOM	65 (69.1)	79 (61.2)
Redness	60 (92.3)	72 (91.1)
Bulging	29 (44.6)	52 (65.8)
Otorrhea	4 (6.2)	8 (10.1)
Bilateral AOM	29 (30.9)	50 (38.8)
Redness	29 (100)	45 (90.0)
Bulging	17 (58.6)	36 (72.0)
Otorrhea	3 (10.3)	5 (10.0)

**Symptoms at baseline (parent-reported), *n* (%)^a^**		
Proportion of children with ear pain	87 (98.9)^b^	117 (97.5)
Proportion of children with fever	40 (48.2)	67 (59.8)

**Oral antibiotics, immediate prescriptions, *n* (%)**	22 (23.4)	48 (37.2)
Amoxicillin	17 (77.3)	45 (93.8)
Amoxicillin/clavulanic acid	1 (4.5)	3 (6.3)
Azitromycin	3 (13.5)	0 (0.0)
Cotrimoxazole	1 (4.5)	0 (0.0)

*Values given as* n *(%) unless stated otherwise. Details on other baseline symptoms are presented in Supplementary Table S1.*

a *Missing data for both intervention (I) and usual care (UC) groups: weight (I,* n *= 19; UC,* n *= 31), daycare attendance (I,* n *= 13; UC,* n *= 21), education father (I,* n *= 26; UC,* n *= 34), education mother (I,* n *= 15; UC,* n *= 26), vaccination (I,* n *= 13; UC,* n *= 20), household smoking (I,* n *= 13; UC,* n *= 21), otorrhea prior to consultation (I,* n *= 16; UC,* n *= 29), ear pain prior to consultation (I,* n *= 11; UC,* n *= 21), fever prior to consultation (I,* n *= 11; UC,* n *= 16), temperature (I,* n *= 4; UC,* n *= 6), ill appearance (I,* n *= 2; UC,* n *= 6), ear pain at baseline (I,* n *= 6; UC,* n *= 9), fever at baseline (I,* n *= 11; UC,* n *= 17).*

b Ear pain as reported by parents, GPs reported ear pain in all subjects. °C = degrees Celsius. AOM = acute otitis media. ENT = ear, nose, and throat. URTI = upper respiratory tract infection.

### Analgesic and OTC medication use

GPs recommended analgesics in 100% of children in the intervention group versus 92.6% of those in the control group (parent reported; Table 2). During the first 3 days, parents in the intervention group gave their children analgesics more frequently compared to those in usual care; the difference was most pronounced for ibuprofen, with almost half of children in the intervention group versus one-tenth in the control group receiving this medication (Table 2). Paracetamol was used in the vast majority of all trial participants initially, but parents in the intervention group gave their child paracetamol for more days, more regularly, and at a slightly higher dosage than those in the control group. (Table 2). Still, overall dosing was lower than recommended by the intervention, as by national guidelines.^4^ Use of nasal drops or spray, analgesic ear drops, and complementary medicine was similar in both groups (parent-reported; see Supplementary Table S2).

**Table 2.** Analgesic use

**Characteristics**	**Intervention (*N*= 94)**		**Usual care (*N*= 129)**	
	***N*^a^**	***n* (%)^b^**	***N*^a^**	***n* (%)^b^**
**Analgesics recommended by GP**	82	82 (100)	108	100 (92.6)
**Analgesics at day of consultation**	88		120	
Paracetamol use		76 (86.4)		103 (85.8)
Doses, *n* ^c^		2.3 ± 1.15		2.1 ± 1.1
Dosage, mg/kg^d^		33.8 ± 19.1		29.0 ± 17.1
Ibuprofen use		39 (44.3)		19 (15.9)^e^
Doses, *n* ^c^		1.8 ± 0.8		1.6 ± 0.9
Dosage, mg/kg^d^		10.4 ± 6.8		12.4 ± 5.9
**Analgesics day 1**	89		116	
Paracetamol use		67 (75.3)		72 (62.2)^e^
Doses, *n*^c^		2.7 ± 1.0		2.3 ± 1.2
Dosage, mg/kg^d^		42.5 ± 20.0		34.2 ± 21.0
Ibuprofen use		46 (51.7)		17 (14.6)^e^
Doses, *n* ^c^		2.4 ± 0.8		2.3 ± 1.0
Dosage, mg/kg^d^		13.2 ± 6.7		18.6 ± 7.6
**Analgesics day 2**	88		117	
Paracetamol use		59 (67.0)		57 (48.8)^e^
Doses, *n*^c^		2.7 ± 0.9		2.1 ± 1.1
Dosage, mg/kg^d^		43.2 ± 20.7		33.0 ± 19.4
Ibuprofen use		38 (43.2)		14 (12.0)
Doses, *n*^c^		2.6 ± 0.8		1.7 ± 0.8
Dosage, mg/kg^d^		14.1 ± 6.3		12.6 ± 8.4
**Analgesics day 3**	86		115	
Paracetamol use		51 (59.3)		41 (35.7)
Doses, *n* ^c^		2.3 ± 1.1		1.9 ± 1.1
Dosage, mg/kg^d^		34.9 ± 20.2		30.6 ± 19.9
Ibuprofen use		29 (33.7)		9 (7.9)^e^
Doses, *n*^c^		2.3 ± 0.9		2.1 ± 0.8
Dosage, mg/kg^d^		12.2 ± 7.0		13.7 ± 8.8

Use of other medication (for example, ear drops, nasal spray, and complementary medicine) is presented in Supplementary Table S2.

a Data available from diary or phone call; primary outcome data were available for all of these patients.

b Unless stated otherwise.

c Mean ± SD, based on children that received ≥1 dose of analgesics.

d Mean ± SD, weight was not reported in 50 children.

e Percentages calculated prior to rounding up.

### Primary outcome

**Figure 2.**
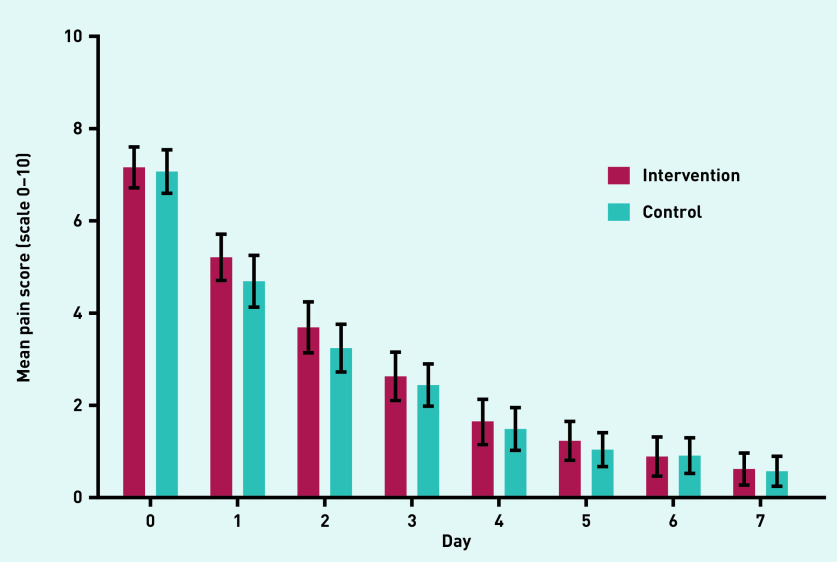
***Mean parent-reported ear pain scores (observed values).***

The observed mean ear pain scores over time are plotted in Figure 2. The mean ear pain score over the first 3 days was similar for children in the intervention and control group (crude MD between groups = 0.16, 95% CI = −0.55 to 0.88; adjusted MD = −0.05, 95% CI = −0.93 to 0.83). Sensitivity analyses, in which missing baseline and outcome variables were imputed, showed similar results (Table 3). Residual analysis showed some deviation from normality as well as some heteroscedasticity. Additional analyses with robust standard errors showed similar results to those observed in the main analysis.

**Table 3.** Primary outcome, mean ear pain score over first 3 days

		**Intervention (*N*= 94)**	**Usual care (*N*= 129)**	**Effect estimate**
**Analysis**	***n***	**Mean (95% CI)**	**Mean (95% CI)**	**Mean difference (95% CI)**
**Intention-to-treat**				
**Over first 3 days**				
Crude	210	4.66 (4.23 to 5.08)	4.36 (3.99 to 4.73)	0.16 (−0.55 to 0.88)
Adjusted^a^	138			−0.05 (−0.93 to 0.83)
**Per day**				
Day 0 (at inclusion)		7.20 (6.66 to 7.73)	7.03 (6.57 to 7.49)	
Day 1		5.15 (4.61 to 5.68)	4.70 (4.23 to 5.16)	
Day 2		3.69 (3.15 to 4.22)	3.27 (2.91 to 3.74)	
Day 3		2.60 (2.06 to 3.14)	2.43 (1.97 to 2.90)	
**Sensitivity analysis: imputation for baseline variables (including confounders)**				
Adjusted^a^	223			0.03 (−0.68 to 0.73)
**Sensitivity analysis: imputation for baseline and outcome variables (including confounders)**				
Adjusted^a^	223			0.01 (−0.78 to 0.79)

a Adjusted for: sex, age, unilateral versus bilateral acute otitis media, ill appearance, number of days ear pain prior to consultation, ear pain at inclusion, otorrhea at inclusion, fever at inclusion, history of recurrent otitis media, oral antibiotic prescriptions (immediate).

### Secondary outcomes

Results for the main secondary outcomes are presented in Table 4.

**Table 4.** Secondary outcomes

		**Intervention (*N*= 94), mean (95% CI)**		**Usual care (*N*= 129), mean (95% CI)**		**Effect estimate, rate ratio (95% CI)**

**Characteristic**	***n***		***n***			
**Days with complaints**						
Ear pain	90	7.58 (6.59 to 8.56)	121	6.76 (5.96 to 7.56)	crude	1.12 (0.94 to 1.34)
					adjusted^a^	1.13 (0.96 to 1.31)
Fever	85	2.98 (2.39 to 3.57)	116	3.37 (2.83 to 3.91)	crude	0.88 (0.68 to 1.14)
					adjusted^a^	0.99 (0.78 to 1.25)

**Oral antibiotic prescriptions**						
At index consultation	94	0.23 (0.15 to 0.31)	129	0.39 (0.30 to 0.48)	crude	0.60 (0.40 to 0.91)
					adjusted^b^	0.66 (0.43 to 1.03)
After index consultation	94	0.19 (0.11 to 0.28)	129	0.09 (0.04 to 0.13)	crude	2.25 (1.09 to 4.62)
					adjusted^b^	2.27 (1.05 to 4.92)
Total	94	0.43 (0.31 to 0.54)	129	0.47 (0.37 to 0.58)	crude	0.90 (0.64 to 1.27)
					adjusted^b^	0.97 (0.68 to 1.38)

**AOM-related GP consultations during 28-day follow-up**						
GP visits only	94	0.63 (0.45 to 0.81)	129	0.36 (0.25 to 0.48)	crude	1.72 (1.12 to 2.65)
					adjusted^a^	1.75 (1.13 to 2.71)
Total (telephone and visits)	94	0.70 (0.51 to 0.89)	129	0.41 (0.29 to 0.54)	crude	1.71 (1.14 to 2.57)
					adjusted^a^	1.73 (1.14 to 2.62)

**Working days lost**						
Father	71	0.17 (0.05 to 0.29)	96	0.29 (0.12 to 0.46)	crude	0.58 (0.22 to 1.53)
					adjusted^a^	0.60 (0.23 to 1.55)
Mother	78	0.37 (0.12 to 0.62)	100	0.49 (0.28 to 0.70)	crude	0.78 (0.36 to 1.62)
					adjusted^a^	0.70 (0.42 to 1.17)

**Daycare or school hours lost**	76	7.2 (4.9 to 9.5)	100	9.1 (6.8 to 11.4)	crude	0.79 (0.52 to 1.19)
					adjusted^a^	0.88 (0.60 to 1.29)

					**Odds ratio (95% CI)**	

**Proportion of children with ear pain, *n* (%)**						
After 24 hours	87	83 (95.4)	118	100 (84.7)	crude	3.74 (1.22 to 11.47)
					adjusted^a^	3.12 (0.91 to 10.65)
After 72 hours	86	63 (73.3)	116	74 (63.8)	crude	1.56 (0.85 to 2.86)
					adjusted^a^	1.37 (0.70 to 2.70)
After 7 days	77	16 (20.8)	97	16 (16.5)	crude	1.33 (0.62 to 2.86)
					adjusted^a^	1.39 (0.57 to 3.41)

					**Mean difference (95% CI)**	

**OM-6 questionnaire^c^ at end of follow-up, mean ± SD**						
OM-6 score	80	2.20 ± 1.07	102	1.97 ± 1.25	crude	−0.23 (−0.57 to 0.12)
					adjusted^d^	−0.18 (−0.51 to 0.15)

**EQ-5D VAS score^e^ after 14 days, mean ± SD**						
Father	66	81 ± 17	95	86 ± 13	crude	4.71 (0.05 to 9.37)
					adjusted^d^	3.90 (−0.08 to 7.88)
Mother	70	83 ± 11	97	83 ± 15	crude	0.19 (−3.91 to 4.29)
					adjusted^d^	−1.38 (−5.14 to 2.38)

a Adjusted for: sex; age; unilateral versus bilateral acute otitis media; ill appearance; number of days ear pain prior to consultation; ear pain; fever and otorrhea at inclusion; history of recurrent otitis media; oral antibiotic prescriptions (immediate).

b Adjusted for all factors previously given in prior footnote excluding oral antibiotic prescriptions (immediate).

c Score on 6-item questionnaire, scale 6–42.

d Additionally adjusted for baseline score.

e Score on a VAS scale ranging from 0–100. AOM = acute otitis media. EQ-5D VAS = EuroQol 5 dimensions visual analogue scale. OM-6 = otitis media-6. SD = standard deviation.

Children in the intervention group received fewer immediate antibiotic prescriptions than those in the control group (adjusted rate ratio [aRR] = 0.66; 95% CI = 0.43 to 1.03), but the total number of antibiotic prescriptions during the 28-day follow-up was similar (aRR 0.97; 95% CI = 0.68 to 1.38). Children in the intervention group reconsulted their GP more often for AOM-related complaints during follow-up (aRR = 1.73; 95% CI = 1.14 to 2.62). The numbers of days with ear pain and fever; proportion of children with ear pain at 24 hours, 72 hours, and 7 days; QoL of children and their parents; days lost from work for parents; and days lost from day-care or school for children were similar in both groups.

In the control group, one child was hospitalised for 5 days because of an acute mastoiditis. This complication occurred 2 days after the index consultation; both paracetamol and ibuprofen were recommended and antibiotics were not prescribed. No (serious) adverse effects of analgesics were reported (data not shown).

In the intervention group, one child was referred to the paediatric emergency department because of persistent fever, despite being prescribed oral antibiotics at the index consultation. Six children in the intervention group were referred to the ENT outpatient department; four for recurrent AOM; one for symptoms of obstructive sleep-disordered breathing; and one for persistent fever. In the control group, one child was referred for recurrent AOM (data not shown).

## DISCUSSION

### Summary

This educational intervention to improve pain management in children with AOM led to an increase in analgesic use, particularly ibuprofen, but did not result in lower parentreported ear-pain scores. Although children in the intervention group received fewer immediate antibiotic prescriptions, the total number of prescriptions over 28 days followup was similar in both groups, which suggests no beneficial effect of this intervention on antibiotic use compared to the consistent findings of trials of delayed prescription where antibiotic use is reduced.^36^^,^^43^^–^^45^ Parents of children in the intervention group reconsulted their GP more often for AOM-related complaints during follow-up than those receiving usual care.

### Strengths and limitations

In contrast to the majority of trials in AOM research, the current study focused on pain management. By applying few exclusion criteria and with median age and reported symptom durations being similar with previous childhood AOM studies in primary care,^46^ the study managed to capture a sample of children with AOM closely mimicking everyday practice. The intervention at trial was tailored to GPs and parents, and developed by a multidisciplinary team to include an online training module, an interactive session, an information leaflet, and analgesic prescription.^18^ Both parentreported outcomes as well as medical records data were collected, which allowed the authors to capture a broad range of subjective and objective outcomes, use a patient-relevant primary outcome, and had few missing data. Information on the severity and duration of the child’s AOM symptoms at baseline allowed the authors to adjust for any differences in illness-severity between groups.

This study shows that an educational intervention, based on accepted clinical practice guidelines, is not necessarily beneficial for the patient, even if the intervention proves effective in changing clinician (prescribing) behaviour. As such, this study highlights the importance of careful evaluation of any intervention before considering introduction, and of carefully assessing the benefits and harms of medicines already widely used in everyday practice.

Despite not reaching the a priori defined sample size due to slower-than-anticipated recruitment, the authors believe that this study is sufficiently powered to draw meaningful conclusions. The target sample size was substantially inflated to account for the cluster design, whereas the random intercept for GP practice was found to be close to zero in this trial. The robustness of the findings was confirmed in the sensitivity analyses, in which missing baseline and outcome data were imputed, but also in the observation that the effect estimates for the primary outcome and associated 95% CIs excluded a difference considered clinically relevant.

The possibility cannot be entirely excluded that this trial might have been subject to post-randomisation differential recruitment, a phenomenon that is sometimes observed in cluster RCTs.^47^ Indeed, more children have been recruited to the control than the intervention group, and the baseline table suggests slight differences between treatment groups. However, no difference was observed between the crude and adjusted analyses, suggesting that this phenomenon did not substantially impact the findings.

### Comparison with existing literature

Although children in the intervention group received paracetamol more regularly, for more days, and at a slightly higher dosage than those in the control group, overall dosing of analgesics was lower than recommended in the intervention and in the clinical practice guidelines.^4^ The same observation was made in trials focusing on effectiveness of antibiotics in children with AOM, in which the role and use of analgesics was discussed with parents as part of the protocol.^36^^,^^40^^,^^48^ This poses the question whether higher dosing of paracetamol by parents is achievable at all. Future qualitative research may unravel reasons and mechanisms for parents’ reluctance to give their children paracetamol in age- or weight-appropriate dosages.

Ibuprofen, when added to paracetamol if pain control was insufficient, had no effect on symptom control; this adds to the current limited, very low quality, evidence failing to detect a benefit of combined use of paracetamol and ibuprofen over paracetamol or ibuprofen alone, with regards to AOM symptoms.^6^

The increase in reconsultations with subsequent antibiotic prescriptions in the intervention group is remarkable. It may well be due to more extensive safetynetting advice in the intervention group. In contrast to the current Dutch guidelines,^4^ which advise reassessment if the child’s condition worsens or if pain and/or fever do not improve after 3 days, the information leaflet advised reconsultation if the child did not recover despite treatment, if the ear pain worsened, or if the ear pain had not resolved after 3 days. Hence, the leaflet may have increased the probability of reconsultation in the intervention group.

Alternatively, parents in the intervention group may also have been more likely to report higher ear pain scores than those in the control group (thereby obscuring an actual decline) leading to more reconsultation, as a result of the very nature of the intervention, which focuses on managing ear pain. The qualitative work with GPs in the intervention group confirmed that they did put an increased focus on treating symptoms with analgesics rather than treating the infection with antibiotics.^49^ Conversely, such an approach might be expected to reduce pain scores due to a placebo effect by recommending greater use and benefit of analgesics.

Arguably, parents from the intervention group may have been more inclined to reconsult their GP when symptoms persisted despite the increase in analgesics use (‘perceived treatment failure’), or owing to children in the intervention group receiving fewer immediate antibiotic prescriptions. The latter, however, is less likely, given the lack of evidence for an effect of immediate antibiotics on symptoms at days 3–7,^50^ and Dutch parents generally accepting analgesics as stand-alone therapy in AOM.^51^

Finally, higher reconsultation rates and subsequent antibiotic prescriptions may have been due to an increased use of ibuprofen. In a post-hoc analysis, children who used ibuprofen were compared with those who did not; children who used ibuprofen had higher pain scores, were more likely to reconsult the GP for AOM-related complaints, and received more antibiotic prescriptions, independent from initial pain scores and treatment allocation. In recent years, evidence is mounting that the use of NSAIDs may be harmful, not just due to the well-known gastric and renal side effects, but due to direct harm on the progression of disease. Limited trial evidence, as well as observational studies, suggest that NSAID use in children and adults with respiratory infections and UTIs leads to a longer duration of symptoms,^10^^,^^11^^,^^13^ an increase in repeat GP consultations,^9^ and a higher risk of complications.^9^^,^^11^^,^^13^^–^^17^ These adverse effects are proposed to be mediated by impaired neutrophil function.^52^^,^^53^ It should, however, be noted that this study’s findings should be interpreted with caution, since confounding by indication cannot be excluded and causality cannot be inferred from the data.

### Implications for research and practice

An intervention targeted at GPs to improve pain management in children with AOM substantially increases analgesic use, particularly ibuprofen, but provides no symptomatic benefit. Future studies should investigate parental barriers to administering paracetamol in age- or weight-appropriate dosage as well as the effectiveness of alternative methods of pain relief in childhood AOM, such as analgesic ear drops. Furthermore, an individually randomised trial of paracetamol (and placebo) versus paracetamol and ibuprofen may now be considered ethical since this study did not find evidence of a beneficial effect of ibuprofen in children with AOM. In the meantime, GPs are advised to carefully weigh the benefits of using ibuprofen in children with AOM against its potential harms.
